# A young female patient with ventricular septal defect complicated with patent foramen ovale complicated with acute cerebral infarction: A case report

**DOI:** 10.1097/MD.0000000000042035

**Published:** 2025-05-23

**Authors:** Jiaqi Wang, Huiliang Liu, Man Gao, Feifei Zhang, Yi Dang, Xiaoyong Qi

**Affiliations:** a Graduate School of Hebei Medical University, Hebei, Shijiazhuang, China; b Cardiovascular Department, Hebei General Hospital, Hebei, Shijiazhuang, China.

**Keywords:** acute cerebral infarction, patent foramen ovale, ventricular septal defect

## Abstract

**Rationale:**

Ventricular septal defect (VSD) is the most common non-cyanotic congenital heart disease. Patent foramen ovale (PFO) is a common benign lesion in healthy people.

**Patient concerns:**

The main symptoms are intermittent dizziness and headache, and weakness in the right limb. Echocardiography showed VSD and PFO, and imaging showed cerebral infarction.

**Diagnoses:**

Typical clinical manifestations, echocardiography found VSD, PFO, and imaging examination suggested cerebral infarction, indicating the diagnosis of VSD, PFO, and old cerebral infarction.

**Interventions:**

Surgical treatment includes closure of VSD and percutaneous closure of PFO, as well as anticoagulation and other related drugs.

**Outcomes:**

Since discharge, the patient’s speech impairment and right limb weakness have returned to normal without any discomfort.

**Lessons:**

We report a patient with VSD complicated with PFO complicated with cerebral infarction. This case highlights the importance of understanding the specific pathological changes in patients with VSD combined with PFO for treatment and prognosis.

## 1. Introduction

Ventricular septal defect (VSD) is the most common non-cyanotic congenital heart disease, affecting 2.62/1000 newborns.^[1]^ Patent foramen ovale (PFO) is a common benign lesion in healthy people and occurs in 25% to 30% of the general population.^[2]^ Recent studies have shown that PFO is closely related to many diseases such as acute coronary syndrome, decompression sickness, cryptogenic stroke, and migraine.^[3]^ PFO is usually asymptomatic, and a small number of patients may have chest pain, headache, and neck pain, which can lead to abnormal embolism, manifested as stroke or peripheral/visceral ischemia.^[4,5]^ Studies have shown that PFO is closely associated with cryptogenic stroke, especially in young people (<55 years).^[6]^ We report a case of VSD complicated with PFO complicated with acute cerebral infarction.

## 2. Case presentation

**Figure 1.**
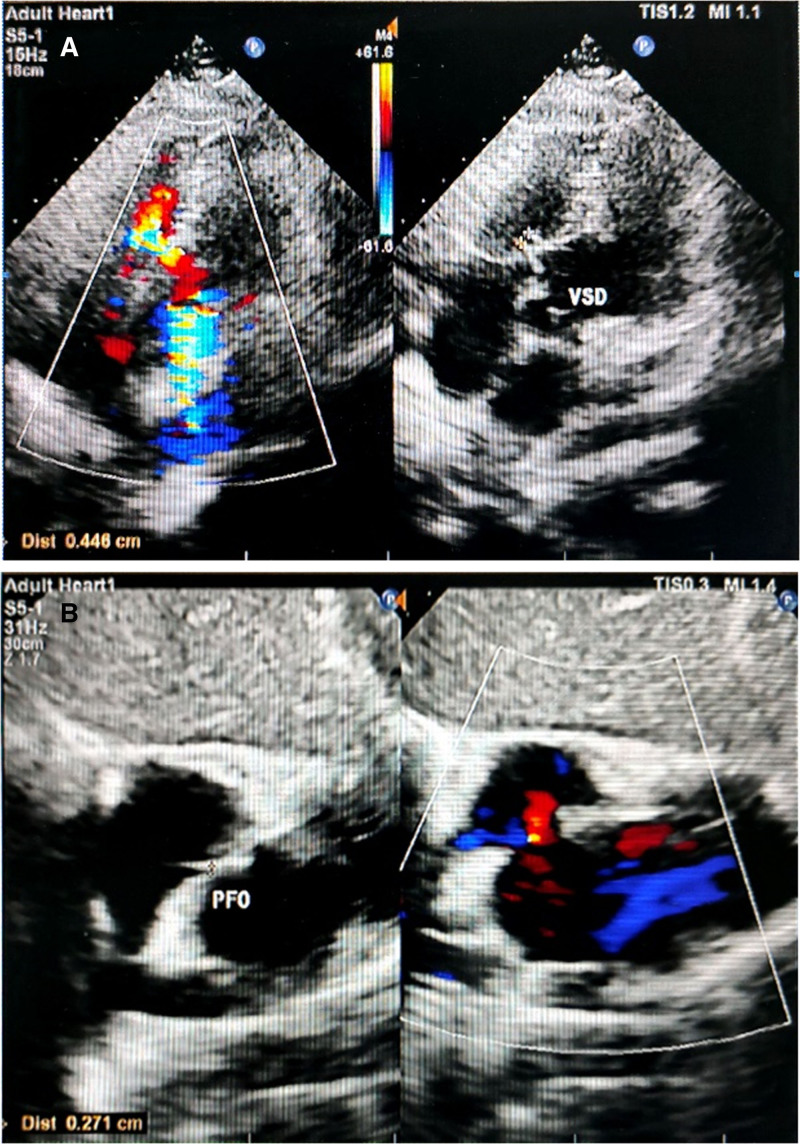
Pre-procedure transthoracic echocardiography of the patient. (A) Ventricular septal defect; (B) patent foramen ovale.

The patient was a 41-year-old female with previously healthy health status. More than 2 months ago, there was no obvious cause of right limb weakness, and she was treated at a local hospital, diagnosed with acute cerebral infarction, and received thrombolytic therapy. Subsequently, the muscle strength of the right limb returned to normal, but dizziness, headache, and heart palpitations still occurred intermittently. Preoperative echocardiography was performed to evaluate in detail the size, location, and relationship of VSDs and PFO to the surrounding cardiac structure. Preoperative transthoracic echocardiography revealed a VSD, PFO, left atrial dilatation, and increased pulmonary artery pressure (Fig. 1A, B). Right heart contrast echocardiography showed (RLS grade III, very large) positive for cTD (grade IV), congenital heart disease, VSD (perimembranous part), and PFO.

**Figure 2.**
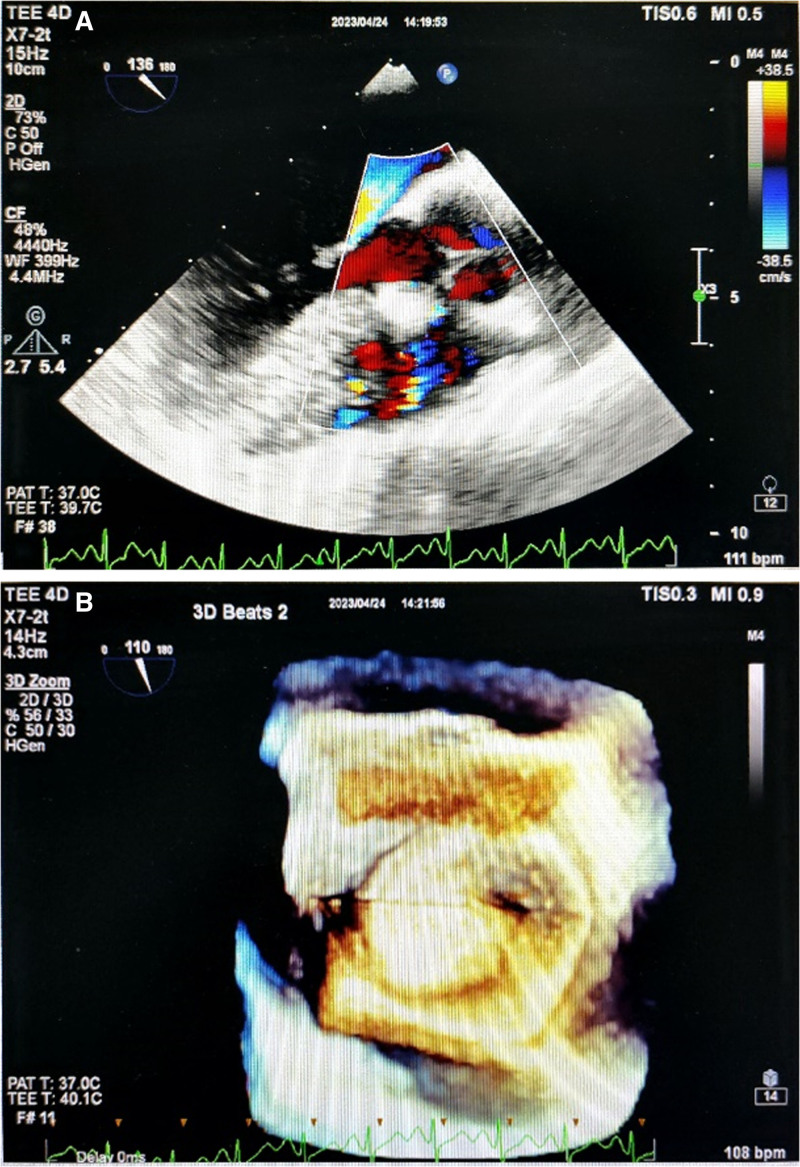
Post-procedure transesophageal 3-dimensional echocardiography (no significant shunt was observed at atrial and ventricular levels).

After admission, the patient was administered oral aspirin for antiplatelet therapy and metoprolol for heart rate control. We chose “percutaneous closure of VSD + left ventricular angiography + ascending aorta angiography + pulmonary venography + percutaneous closure of PFO.” Ascending aortogram showed no aortic regurgitation. Intraoperative left ventricular angiography showed the septal membrane, the left ventricular surface defect was approximately 8.5 mm, and the right ventricular surface defect was approximately 3.7 mm. SFPQ7F and Lifetech 6 mm occlusal parachute were selected for VSD closure. We selected a PF 25 mm umbrella for closure of the PFO. After the operation, the patient was administered low-molecular-weight heparin anticoagulation, aspirin, and clopidogrel antiplatelet therapy. Transesophageal 3-dimensional echocardiography showed no obvious shunt in the atria or ventricles (Fig. 2A, B).

The patient had sudden onset of right limb weakness and numbness at night on the first day after surgery. Therefore, a detailed neurological examination and evaluation of the patient is required.^[7]^ Nervous system physical examination: Shen Qing speech, higher cortex function is normal. Bilateral pupils are round and equal, with a diameter of about 3.0 mm, sensitive to light reflex, flexible movement of both eyeballs in all directions, no diplopia and nystagmus, and physical examination of cranial nerves (−). Right limb muscle strength level 4+, left limb muscle strength level 5. Muscle tone in the extremities is normal. The pain of bilateral acupuncture is symmetrical. The pain of acupuncture in the right hand is hypersensitive, and the pain of acupuncture in the other 2 limbs is roughly symmetrical. The right upper limb fingernose test and the right lower limb heel knee shin test were not stable and accurate. The left upper limb fingernose test and left lower limb heel knee shin test were stable and accurate. Tendon reflexes exist symmetrically. Right Babinski sign positive, left Babinski sign negative. Head DWI + MRA: multiple acute or subacute cerebral infarctions in the right frontoparietal insula, left frontoparietal insula, and left basal ganglia. Combined with the patient’s clinical symptoms, nervous system physical examination and imaging examination, the diagnosis of cerebral infarction was confirmed.^[8]^ After neurological consultation, edaravone and butylphthalide were recommended for the treatment of cerebral infarction. The patient suddenly developed speech impairment on the 4th day after surgery, and head CT examination showed no obvious bleeding. Head DWI reexamination showed multiple limited signals in both cerebral hemispheres, and the lesions in the left lateral ventricle and left basal ganglia were larger than before. The patient underwent physical examination with clear mind and poor speech; the right nasolabial ditch was slightly shallower than the left; the mouth Angle was not crooked; the muscle strength of the right lower limb was grade IV; the right rotation test was less flexible than the left; the right finger nose test and the heel knee and shin test were less stable; the right Babinski sign and bilateral Chaddock sign were positive; and no obvious abnormalities were found in the physical examination of the remaining nervous system.

## 3. Discussion

According to the different defect sites, VSD can be divided into 4 types: perimembranous, muscular, outflow tract, and inflow tract. Among them, perimembranous VSD is the most common, accounting for approximately 80% of all VSD.^[9]^ Percutaneous VSD closure has the advantages of a high success rate and low complication rate, and is widely used in the treatment of VSD.^[10]^ Although PFO accounts for 25% of normal adults,^[6]^ VSD combined with PFO is rare. Wahl et al 10-year follow-up of 308 patients with PFO who were treated with closure, anticoagulation or antiplatelet therapy showed that closure significantly reduced the incidence of TIA and stroke.^[11]^ Therefore, when an infarction of undetermined cause, transesophageal echocardiography should be routinely performed to look for possible causes of embolism, and PFO closure and antiplatelet aggregation or anticoagulation therapy should be given simultaneously to reduce the occurrence of arterial thrombosis.^[12]^ The European Stroke Organization recommends that PFO closure is superior to medication for patients aged 18 to 60 years who have a cryptogenic stroke and are at high risk for PFO with moderate or severe shunt, atrial septal aneurysm, or atrial septal hyperactivity.^[13]^ In this case, PFO and VSD were actively closure, and dual antiplatelet and anticoagulant therapy was given after surgery, and good therapeutic effect was finally achieved. The patient had a history of cerebral infarction caused by paradoxical embolism at the atrial level 2 months before surgery, and a new cerebral infarction after surgery. The possible cause was blood flow in the right atrial surface of the foramen ovale caused by repeated release of the occluding umbrella during foramen ovale occlusion, or fresh thrombus between the 2 discs of the occluding device entered the left cardiac system, leading to a new cerebral infarction. Therefore, in clinical practice, foramen ovale closure should be performed with more caution. For patients with unsmooth intraoperative release of the occlusive device and multiple releases, the changes of the patient’s condition should be closely observed during the perioperative period, and suspicious complications should be dealt with as soon as possible to reduce new damage caused by medical complications.

## 4. Conclusion

PFO with VSD is rare. Further studies are needed to safely and effectively prevent cerebral infarction in such patients. A comprehensive and accurate understanding of PFO with VSD and its underlying pathophysiological mechanisms is of great significance for improving patient prognosis.

## Acknowledgments

We thank the patient and her family for giving us permission to share this rare case, and express our gratitude to the imaging department and ultrasound department for providing us with imaging data.

## Author contributions

**Formal analysis:** Huiliang Liu.

**Investigation:** Man Gao, Feifei Zhang.

**Methodology:** Xiaoyong Qi.

**Software:** Huiliang Liu, Xiaoyong Qi.

**Validation:** Yi Dang.

**Writing – original draft:** Jiaqi Wang.
